# Preparatory attention to visual features primarily relies on non-sensory representation

**DOI:** 10.1038/s41598-022-26104-2

**Published:** 2022-12-16

**Authors:** Mengyuan Gong, Yilin Chen, Taosheng Liu

**Affiliations:** 1 Department of Psychology and Behavioral Sciences, Zhejiang University, Hangzhou, 310028 People’s Republic of China; 2 Department of Psychology, Michigan State University, East Lansing, Michigan 48824 USA

**Keywords:** Cognitive neuroscience, Attention

## Abstract

Prior knowledge of behaviorally relevant information promotes preparatory attention before the appearance of stimuli. A key question is how our brain represents the attended information during preparation. A sensory template hypothesis assumes that preparatory signals evoke neural activity patterns that resembled the perception of the attended stimuli, whereas a non-sensory, abstract template hypothesis assumes that preparatory signals reflect the abstraction of attended stimuli. To test these hypotheses, we used fMRI and multivariate analysis to characterize neural activity patterns when human participants were prepared to attend a feature and then select it from a compound stimulus. In an fMRI experiment using basic visual feature (motion direction), we observed reliable decoding of the to-be-attended feature from the preparatory activity in both visual and frontoparietal areas. However, while the neural patterns constructed by a single feature from a baseline task generalized to the activity patterns during stimulus selection, they could not generalize to the activity patterns during preparation. Our findings thus suggest that neural signals during attentional preparation are predominantly non-sensory in nature that may reflect an abstraction of the attended feature. Such a representation could provide efficient and stable guidance of attention.

## Introduction

We are surrounded by a plethora of sensory information in our environment, which necessitates attentional selection for task-relevant information^1,2^. Oftentimes, we also know what we are looking for before the arrival of the task-relevant target. The brain is known to address this challenge by biasing competition in favor of the target stimulus during preparatory periods^3,4^. Previous neuroimaging studies have shown that preparatory attention toward different feature dimensions (e.g., color vs. motion) selectively increases activity in corresponding visual areas before the stimulus onset^5–7^. However, how preparatory attention to feature values (e.g., leftward vs. rightward motion) in human brain is represented remains largely unknown.

A number of studies have shown that preparatory attention to familiar objects evokes brain activities that resemble those during the perception of the same stimulus, which we refer to as a “sensory template^8–10^”. Other studies in the literature, however, showed that attention can tune neural response to high-level information such as abstract categories^11–13^, and also suggested flexible re-coding of sensory information into distinct formats during retention of mnemonic contents^14,15^. This latter work thus raised an alternative possibility of non-sensory representation during preparation for to-be-attended features. Although these studies supporting the latter account often did not specifically focus on preparatory attention and they also tended to target different brain areas than studies supporting sensory templates (visual areas vs. frontoparietal areas), they provide contrasting theoretical perspectives on the nature of neural representations for preparatory attention.

We thus set out to examine whether preparatory activity (during the anticipation of upcoming visual stimuli) reflects representations of attended feature in sensory or non-sensory formats, using functional magnetic resonance imaging (fMRI). In an attention task, participants were pre-cued about a task-relevant feature (motion direction) in a subsequent display. After a delay period, they were shown a compound stimulus with two overlapping features. In a baseline task, we presented a single feature stimulus and used it to measure the neural representation of the physical features (sensory template). We then used multivoxel pattern analysis (MVPA) to assess the neural ensemble representation for the to-be-attended feature in the preparatory period and its relationship to the sensory template. A *sensory template hypothesis* predicts that attentional modulation of the preparatory activity primarily reflects the perception of a single direction stimulus in the baseline task, whereas *a non-sensory, or abstract template hypothesis* predicts decodable neural patterns during preparation, which, however, does not necessarily reflect sensory properties of the stimulus. Our results showed that preparatory activity contained information of the to-be-attended feature, but its representational format likely reflects a non-sensory abstraction of low-level features that then transforms into a sensory format during stimulus selection.

## Methods

### Participants

Twelve individuals (6 females, mean age = 27.6) from Michigan State University participated in the main fMRI experiment. Another group of twelve individuals (5 females, mean age = 21.3) from Zhejiang University participated in a control experiment. The sample size was comparable to previous studies using similar attention tasks^11,16–19^. One participant from the fMRI experiment was left-handed and all others from the two experiments were right-handed, they had a normal or corrected-to-normal vision. Participants provided written informed consent according to the study protocol approved by the Institutional Review Board at Michigan State University and Zhejiang University. The research was conducted in accordance with the 7th revision of the Declaration of Helsinki 2008. They were paid $20 per hour for their participation in the main experiment and ¥30 for their participation in the control experiment.

### Stimuli and apparatus

#### Main fMRI experiment.

Stimuli were generated using MGL^20^, a set of custom OpenGL libraries implemented in MATLAB (The MathWorks, Natick, MA). The stimuli were presented on a CRT monitor (resolution: 1024 × 768, refresh rate: 60 Hz) during behavioral training, at a viewing distance of 91 cm in a dark room. During the fMRI scans, stimuli were projected on a rear-projection screen located in the scanner bore by a Hyperion MRI Digital Projector (Psychology Software Tools, Sharpsburg, PA). The resolution and refresh rate were the same as the CRT monitor. Participants viewed the screen via an angled mirror attached to the head coil at a viewing distance of 60 cm.

The stimulus aperture was an annulus with an inner radius of 1.5° and an outer radius of 6°, centered on the fixation cross against a dark background. There were two types of stimuli: two superimposed dot fields (dot size: 0.1°, density: 2.5 dots/degree2, speed: 2.5°/s) that moved along the leftward (~ 135°) and rightward (~ 45°) directions; a single dot field that moved along one of the two directions (~ 135° or ~ 45°). When a dot moved out of the aperture, it was wrapped around to reappear from the opposite side along its motion direction.

#### Control experiments

Stimuli were generated using Psychtoolbox^21,22^ implemented in MATLAB. The stimuli were presented on a 17-inch CRT monitor (resolution: 1024 × 768, refresh rate: 100 Hz) at a viewing distance of 60 cm in a dark room. The parameters of the stimulus were identical to that used in the main experiment.

### Experimental design and statistical analysis

#### Overall procedures in the fMRI experiment

Each participant completed a behavioral training session and a subsequent scanning session on different days. We used the training session to familiarize participants with the behavioral task and also calibrated their performance using a staircase procedure (Best Parameter Estimation by Sequential Testing, Best PEST), as implemented in the Palamedes Toolbox^23^. During training, participants completed at least 3 blocks (30 trials/block) of the task to obtain an estimate of their threshold, which was used for both the attention and baseline tasks in the scanning session to equate the sensory input between tasks. Each participant completed 6 runs (30 trials/run) of the attention task and 2–3 runs (61 trials/run) of the baseline task in the scanner.

#### Main fMRI experiment: Motion-based attention and baseline task

**Figure 1:**
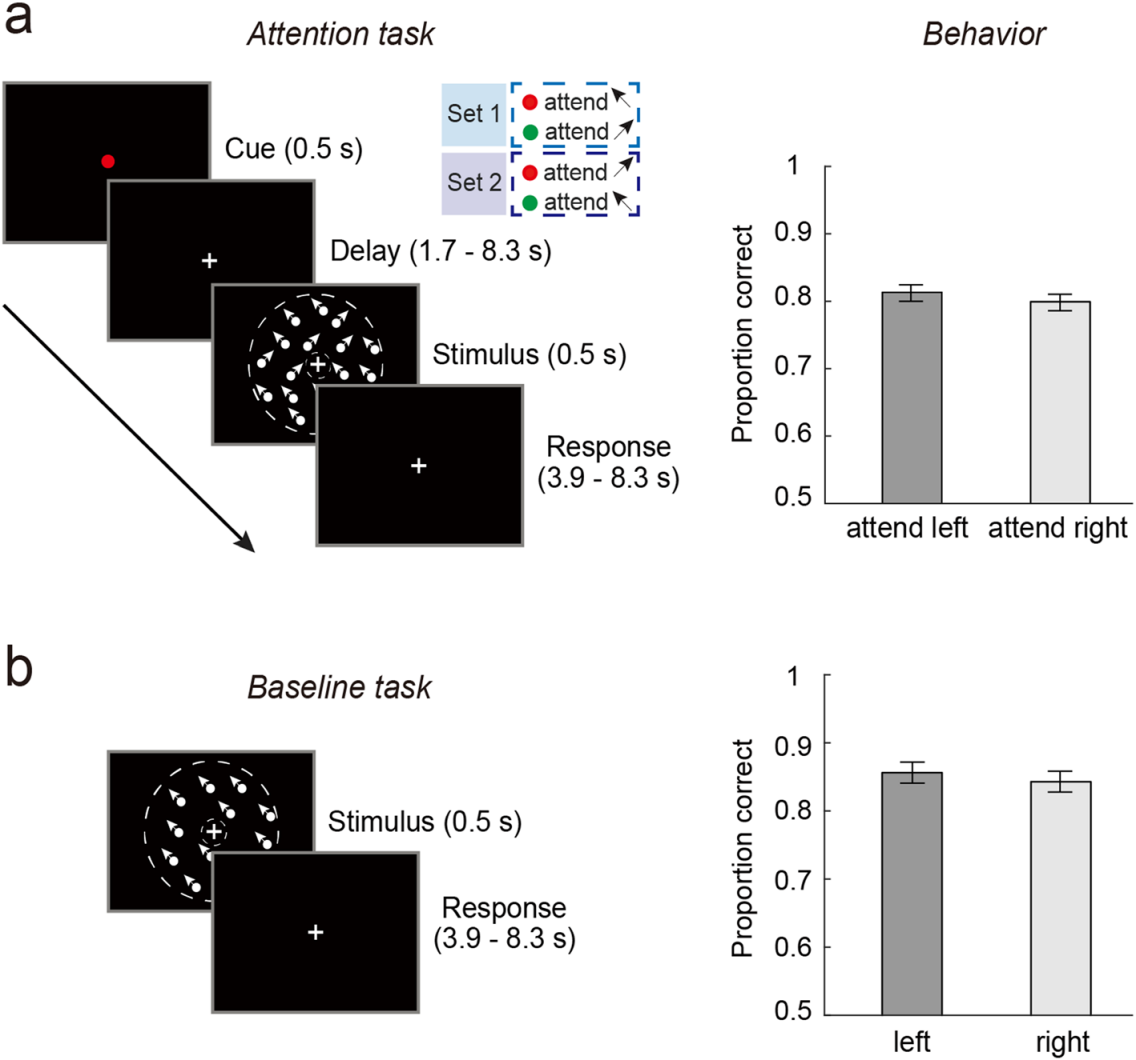
(**a**) The schematic of the motion-based attention task and behavioral accuracy in this task. The cue color indicated the attended motion direction (leftward vs. rightward), followed by a delay period and a compound stimulus comprised of two superimposed moving dot fields. Two sets of mappings between the cue color and attended direction were used in separate runs (indicated by the inset). The gray circles demarcate the annulus-shaped aperture; they were not shown in the actual stimuli. (**b**) The schematic of the motion-based baseline task and performance in this task. A single moving dot field along leftward or rightward direction was shown, followed by a response display. Error bars are within-subject standard errors, using the method by Cousineau^24^.

On each trial of the attention task (Fig. 1A, left panel), a color cue was presented for 0.5 s to indicate the to-be-attended direction (leftward or rightward) with 100% validity, followed by an inter-stimulus interval varying from 1.7 s to 8.3 s with different probabilities (10% for 1.7 s or 3.9 s each, 40% for 6.1 s or 8.3 s each). The majority of trials (80%) were of long delays (6.1 s or 8.3 s) to allow the separation of the preparatory activity from the stimulus-evoked response during fMRI scanning. Then, two superimposed dot fields moving in two directions were shown for 0.5 s, followed by an inter-trial interval of 4.4 s to 8.8 s (2.2 s per step). The attended direction moved along the diagonal direction (e.g., 135°) with a small angular offset, whereas the unattended direction moved along the diagonal direction (e.g., 45°). The angular offset was adjusted by Best PEST in the training session prior to the scanning to obtain a threshold that corresponded to a performance level of ~ 75%. This threshold was then used as a constant offset in the scanning session. Participants used a keypad to report whether the attended motion direction moved more clockwise (CW) or counterclockwise (CCW) relative to the reference diagonal direction. We provided trial-by-trial feedback (“correct” or “incorrect”) in the training session and the percentage of correct responses at the end of each run in the scanning session to avoid the influence of trial-level feedback on neural activity.

Note that we did not include trials with neutral or invalid cues because of the limit in scanning time. However, our previous study using a similar feature cueing paradigm found a benefit of preparatory attention on behavioral performance when comparing valid and neutral conditions^25^. To prevent the cue-related sensory difference from contributing to neural activity, we reversed the mapping between colors and directions halfway through the experiment. Participants were informed about the association between the cued color (red and green) and attended direction (leftward and rightward) at the beginning of each run, such that red indicated “attend to leftward direction” and green indicated “attend to rightward direction” in the first half of the runs, and vice versa for the second half of the runs. The order of the two types of associations was counter-balanced across participants.

On each trial of the motion baseline task (Fig. 1A, right panel), a single dot field was shown for 0.5 s, followed by an inter-trial interval between 3.9 s and 8.3 s. To equate the sensory input between attention and baseline tasks, the motion direction was shifted away from the reference diagonal direction by the same offset as that used in the attention task with each individual participant’s own threshold. Participants performed the same direction discrimination task by reporting whether the moving dots were more CW or CCW relative to the reference diagonal direction. We provided the feedback in the same way as that in the attention task.

#### Control experiments: articulatory suppression

This experiment was conducted to examine whether participants translated information about the cued feature into a verbal format during the preparation period. The trial structure was modelled after the main experiment. Importantly, different from the main experiment, in half of the trials, participants were shown three digits at the beginning of the trial. These digits were randomly selected without replacement from the integers 1–9 and were shown 2° to the left and right, and in the middle of the screen. To disrupt potential verbal re-coding, participants were instructed to perform an articulatory suppression task by repeating aloud out the digits during the preparation period (at approximately 3 digit/sec). In the other half of the trials, participants were shown with three letters (“xxx”) at the beginning of the trial, indicating no need for articulatory suppression and participants remained silent. Note that we intentionally used three, rather than two digits as commonly used in previous studies^26^, to increase the verbal load to detect any interference effect. These two types of trials were randomly interleaved. The experimenter was inside the room to ensure that the participants performed as instructed.

Each participant completed a staircase task and an attention task within a single session. We used the staircase task to familiarize participants with the direction discrimination task and calibrated their performance with Best PEST (at performance level of ~ 75%). Participant completed 2 blocks (40 trials/block) of the staircase task to obtain an estimate of their threshold, which was used in the attention task. Each participant completed 4 runs (32 trials/run) of the attention task.

### Eye tracking

To evaluate the stability of visual fixation, we monitored each participant’s eye position during the training session of the main fMRI experiment at a sampling rate of 500 Hz. We used an Eyelink 1000 system (SR Research, Ontario, Canada). The data were analyzed offline using custom Matlab code.

### fMRI data acquisition

Imaging was performed on a GE Healthcare 3 T Sigma HDx MRI scanner, equipped with an eight-channel head coil, in the Department of Radiology at Michigan State University (East Lansing, Michigan). For each participant, high-resolution anatomical images were acquired using a T1-weighted magnetization-prepared rapid-acquisition gradient echo sequence (field of view, 256 × 256 mm; 180 sagittal slices; 1 mm^3^ resolution). Functional images were acquired using a T2*–weighted echo planar imaging sequence comprised of 30 slices (repetition time / TR 2.2 s; echo time/ TE, 30 ms; flip angle, 78°; matrix size, 64 × 64; in-plane resolution, 3 × 3 mm; slice thickness, 4 mm, interleaved, no gap). In each scanning session, we also acquired a 2D T1-weighted anatomical image that had the same slice prescription as the functional scans but with higher in-plane resolution (0.75 × 0.75 × 4 mm). This image was used to align the functional data to the high-resolution anatomical images for each participant.

### Retinotopic mapping

For each participant in the main fMRI experiment, we ran a separate scanning session to obtain retinotopic maps in visual and parietal areas. Participants viewed four runs of rotating wedges (i.e., clockwise and counterclockwise) and two runs of rings (i.e., expanding and contracting) to map the polar angle and radial components, respectively^27–29^. Borders between areas were defined as the phase reversals in a polar angle map of the visual field. Phase maps were visualized on computationally flattened representations of the cortical surface, which were generated from the high-resolution anatomical image using FreeSurfer (http://surfer.nmr.mgh.harvard.edu) and custom Matlab code.

To help identify the topographic areas in parietal areas, we ran 2 runs of memory-guided saccade task modeled after previous studies^30–32^. Participants fixated at the screen center while a peripheral (~ 10° radius) target dot was flashed for 500 ms. The flashed target was quickly masked by a ring of 100 distractor dots randomly positioned within an annulus (8.5–10.5°). The mask remained on screen for 3 s, after which participants were instructed to make a saccade to the memorized target position, then immediately saccade back to the central fixation. The position of the peripheral target shifted around the annulus from trial to trial in either a clockwise or counterclockwise order. Data from the memory-guided saccade task were analyzed using the same phase encoding method as the wedge and ring data.

In addition, one run consisted of alternating moving versus stationary dots was used to localize the motion-sensitive area, MT + , an area near the junction of the occipital and temporal cortex^33^. Therefore, the following regions of interest (ROIs) in each hemisphere were identified after the completion of this session: V1, V2, V3, V3A/B, V4, V7, MT + , IPS1 to IPS4. Due to the small anatomical size of the IPS sub-regions, we combined V7/IPS0, IPS1 and IPS2 to form a posterior IPS (pIPS), and combined IPS3 and IPS4 to form anterior IPS (aIPS), according to their functional similarities^32^.

### fMRI data preprocessing

Data analyses were performed using mrTools (http://gru.standford.edu/mrTools) and custom code in Matlab. The analyses for the two experiments are presented together as they were nearly identical except minor adjustment of a few parameters, which are noted below. For each run, functional data were preprocessed with head motion correction, linear detrending and temporal high pass filtering at 0.01 Hz. Data were converted to percentage signal change by dividing the time course of each voxel by its mean signals in each run. We concatenated 6 runs of the attention task and the remaining runs of the baseline task (2–3 runs) separately for further analysis.

### Univariate analysis

#### Deconvolution

For the attention task data in each experiment, we used a deconvolution approach by fitting each voxel’s time series with a general linear model (GLM) with ten regressors, four corresponding to the long delay (6.1 s and 8.3 s) trials with correct responses (2 attended directions × 2 delays), two corresponding to the short delay (1.7 s and 3.9 s) trials with correct responses, and the remaining regressor corresponding to incorrect trials for each length of the delay. Each trial was modelled by a set of twelve finite impulse response functions (FIR) after the trial onset. For the baseline task data, we used a GLM with two regressors, corresponding to two features (leftward vs. rightward). Each trial was modelled by a set of eight FIR after the trial onset. The design matrix was pseudo-inversed and multiplied by the time series to obtain an estimate of the hemodynamic response (HRF) evoked by each condition. Correct trials from the attention task and all trials from the baseline task were entered into further univariate and multivariate analysis.

For each voxel, we computed the goodness of fit measure (*r*^2^ value), corresponding to the amount of variance explained by the deconvolution model^34^. The *r*^2^ value represents the degree to which the voxel’s response over time is correlated with the task events, regardless of any differential responses among conditions. For each subject, we defined two frontal areas in each hemisphere that were active during the attention task: one is located superior to the precentral sulcus and near the superior frontal sulcus (FEF) and the other is located towards the inferior precentral sulcus, close to the junction with the inferior frontal sulcus (IFJ).

#### Voxel selection

To select active voxels during the task, we first removed noisy voxels with responses larger than 10% signal change at any time point in the time series. We then used the *r*^2^ values from the deconvolution analysis to index the relevance of each voxel to the task by sorting voxels in each brain area in descending order of their *r*^2^ values. We selected the minimal number of voxels across subjects and ROIs, separately for each experiment. This led to the inclusion of top-ranked 70 voxels for all further analyses. We also performed the analyses using fewer and more voxels, and the results remained highly similar (see Supplementary Information and Figure. S1 online for details).

#### Attentional modulation on BOLD response over time

To assess whether feature-based attention modulated overall BOLD response during the preparatory and stimulus periods across time points, we averaged the fMRI response across voxels for long delay trials in the attention task, separately for each ROI, each attention condition and each time point. Then, we averaged a time window of 2 to 3 TRs after the trial onset for the preparatory activity, and averaged a time window of 2 to 3 TRs after the stimulus onset for the stimulus-evoked response, to obtain a measure of the BOLD response amplitude in these periods. To examine whether attention modulates univariate response during these two periods, we applied separate paired t-tests on the BOLD response amplitudes.

#### Baseline shift index (BSI) during the preparation

To assess whether preparatory attention induces shifts of pre-stimulus neural activity, and how such shifts varied across brain areas, we calculated the baseline shift index^6^ (BSI) for each subject and each ROI, which was defined as the ratio of the response amplitudes during the preparation and stimulus period. Larger BSI values indicate proportionally stronger pre-stimulus activity. To obtain a reliable estimate of the response amplitude while considering the regional variability, we extracted the maximal response based on the averaged response across two attention conditions for each ROI within a broader time window (preparation periods: 1 to 3 TRs after the trial onset; stimulus periods: 1 to 3 TRs after the motion onset). We then applied a one-sample t-test that compares BSI to zero. To examine whether the shifts of baseline differ across cortical areas, we performed one-way repeated-measures ANOVA (10 brain areas) on the BSI. Note we used a slightly different method to extract response amplitude here to avoid extreme values in individual subject data when calculating the ratio, but the results remained qualitatively the same when using the same time window as above.

### Multivoxel pattern analysis (MVPA)

#### Decoding the attended features

To test if multivariate patterns of activity represent information of the attended feature, we conducted separate MVPA on the activity patterns for the preparation and stimulus periods. For this analysis, we extracted fMRI signals from raw time series in the long delay trials with correct behavioral responses (~ 56 trials per attention condition). We excluded short delay trials as they could not provide enough data points to measure preparatory activity, as well as incorrectly responded trials which could be caused by misallocated attention (although including the incorrect trials in the analysis did not change the results qualitatively). We than obtained averaged BOLD response for each voxel and each trial in a given ROI, separately for preparatory activity (2 to 3 TRs after the trial onset) and stimulus-evoked activity (2 to 3 TRs after the stimulus onset). The response amplitudes across two attention conditions in each ROI were further z-normalized, separately for the preparation and stimulus-related activity. These normalized single-trial BOLD responses were used for the MVPA. We trained a classifier using the Fisher linear discriminant (FLD) analysis to discriminate between two attention conditions (leftward vs. rightward) and tested its performance with a leave-one-run-out cross-validation scheme. This process was repeated until each run was tested once and the classification accuracy (i.e., the proportion of correctly classified trials) was averaged across the cross-validation folds. The statistical significance of classification accuracy was evaluated by comparing it to the chance level obtained from the permutation test (see Permutation test).

#### Cross-task generalization from the baseline to attention task

To test the sensory template hypothesis, we used MVPA to determine whether neural patterns in the preparatory and stimulus periods from the attention task reflected a sensory representation of the attended feature alone, as measured by the baseline task with isolated features (i.e., leftward and rightward feature). We trained an FLD classifier using the normalized BOLD responses from the baseline task (2 to 3 TRs after the trial onset) to discriminate leftward vs. rightward feature. We then tested this classifier on the normalized response from the attention task to discriminate between attend leftward vs. attend rightward condition, separately for preparatory activity and stimulus-evoked activity. Because the training and testing data were obtained from two different tasks in separate runs, a cross-validation scheme was unnecessary for evaluating the classifier’s performance. The significance of classification accuracy was compared to the chance level obtained from a permutation test (see Permutation test). To assess if the generalization performance differed between preparation and stimulus periods, we performed a two-way repeated-measures ANOVA (2 time periods × 10 brain areas) on the classification accuracy.

### Control analyses

We performed two analyses to assess if shifts in spatial attention, either overt or covert, occurred during the preparatory period in the attention task.

#### Analysis of overt attentional shift

We first examined the possibility of overt attention during the preparation period. We analyzed the participants’ eye positions recorded during the training session, separately for the horizontal and vertical eye positions. If participants adopted a space-based strategy, we should expect that in attend-to-leftward trials, participants may have directed their gaze leftward, and vice versa for the attend-to-rightward trials. To test this possibility, we applied separate paired t-tests on horizontal and vertical eye positions.

#### Analysis of covert attentional shift

We next examined if there was a shift of covert attention during the preparation period, in which case we may expect attentional modulation on topographically specific BOLD responses. For example, in attend-to-leftward trials, participants may have directed their spatial attention to the upper-left quadrant (or the upper-left and lower-right quadrants along the left diagonal direction), eliciting a stronger response in these attended locations than the unattended quadrants, and vice versa for attend-to-rightward trials (Fig. 4C). To evaluate this possibility, we first determined the response field location of voxels in V1 by calculating the radial and polar angle component of each voxel, using data from the retinotopy session. This allowed us to sort the voxels into four quadrants within the range of the stimulus aperture (1.5–6°). We thus calculated the univariate BOLD activity within each quadrant representation. If participants attended to the upper visual field, we should observe attentional modulations on voxel responses from the two upper quadrants (upper-left and upper-right). If participants attended along the diagonal direction during preparation, we should observe attentional modulations on voxel responses from all four quadrants. To test these possibilities, we grouped two or four quadrants into attended versus unattended locations. We then applied a separate paired t-test on V1 response.

#### Bayesian analyses

To evaluate the strength of evidence for the null hypothesis, which is particularly useful for validating the control analyses above, we conducted Bayesian analyses using JASP^35^. For both of the above control analyses, we conducted Bayesian paired *t*-tests in parallel to the frequentist *t*-tests above. We reported the Bayes factor (BF_01_), which quantifies evidence for the null hypothesis^36,37^.

### Permutation test

For each brain area, we evaluated the statistical significance of the observed classification accuracy using a permutation test scheme. We first shuffled the trial labels in the training data and trained the same FLD classifier on the shuffled data. We then tested the classifier on the test data to obtain classification accuracy. For each ROI and each participant, we repeated this procedure 1000 times to compute a null distribution of classification accuracy. To compute the group-level significance, we averaged the 12 null distributions to obtain a single null distribution of 1000 values for each ROI. To determine if the observed classification accuracy significantly exceeds the chance level, we compared the observed value to the 95 percentiles of this group-level distribution (corresponding to *p* = 0.05). Note that these ROIs were pre-defined with strong priors as their activation in attention tasks has been consistently reported in the literature. Nevertheless, for those analyses where multiple comparisons were needed across brain areas, we applied a false discovery rate (FDR) to adjust the *p*-values^38^.

## Results

### Behavioral performance in main fMRI experiment

Participants were instructed to discriminate a small angular offset. Individually-adjusted thresholds were used for the scanning session (mean offset = 9.2°; SD = 3.8°). As shown in Fig. 1, during scanning, participants’ discrimination accuracy was comparable between the attention and baseline tasks for each experiment (paired *t*-test: *t*(11) = 1.71, *p* = 0.115, BF_01_ = 0.89). No reliable difference was observed between the two attended features (*t*(11) = 0.57, *p* = 0.578, BF_01_ = 3.02). Similar results were obtained between two features in the baseline task (*t*(11) = 0.43, *p* = 0.677, BF_01_ = 3.21). These results indicated comparable level of task difficulty between tasks (attention vs. baseline) and between features (leftward vs. rightward).

### Elevated preparatory activity for attention to motion directions

**Figure 2:**
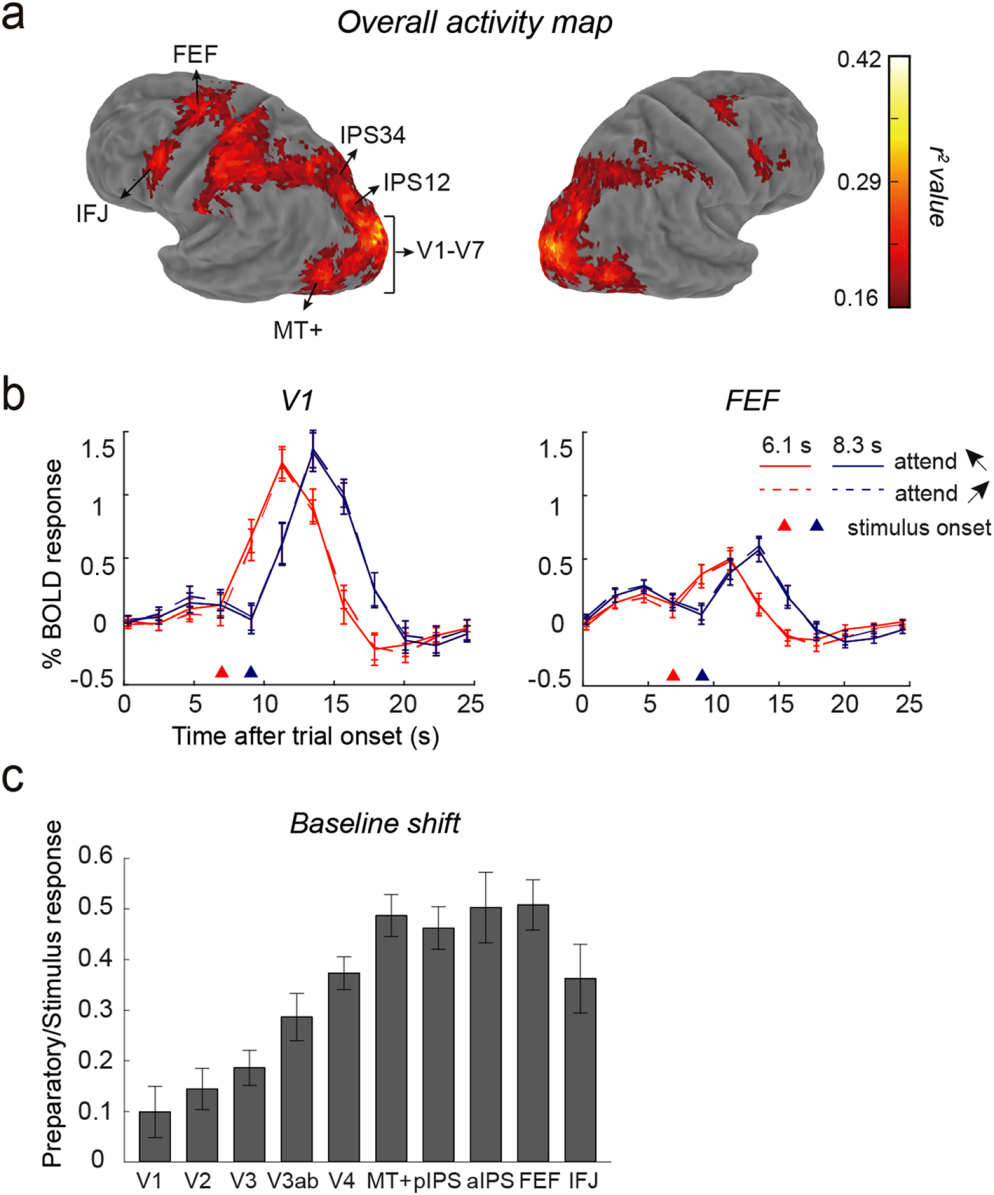
(**a**) Group-averaged *r*^2^ map shown on an inflated Caret atlas surface. The map was thresholded at *r*^2^ value of 0.16 with a cluster constraint of 50 voxels. (**b**) Mean fMRI time course from two representative areas (V1 and FEF). In each plot, the red and blue triangles above the x-axis denote the stimulus onset in the two long-delay trials. (**c**) Baseline shift index (BSI) for all brain areas. BSI was defined as the ratio of the peak response for preparation to stimulus. Error bars denote SEMs. Baseline shifts were significantly larger than zero in all areas (maximal *p* < 0.016, FDR-corrected), except in V1 (*p* = 0.236, FDR-corrected).

Consistent with the literature^39,40^, we found that attending to a particular feature activated the visual cortex and dorsal frontoparietal network. Group-averaged fMRI time courses showed a small, elevated activity during preparation, followed by a robust response to the two superimposed features after its onset (indicated by the triangles, Fig. 2B). There was no difference in mean BOLD response amplitude between attention conditions during either the preparation period (*ps* > 0.794; paired t-tests, *FDR-corrected;* BF_01_ = 2.14 ~ 3.37) or the stimulus period (*ps* > 0.302; paired *t*-tests, *FDR-corrected;* BF_01_ = 1.29 ~ 3.47) in any of the tested brain areas. Thus, mean BOLD response did not exhibit features specificity, consistent with previous work^13,16,41,42^.

The overall level of pre-stimulus activity became more pronounced in higher-tier cortical areas when preparing to attend particular motion directions or oriented gratings. To quantify the magnitude of this elevated response across regions, we calculated a BSI for each area (see Materials and Methods section) for each dataset, which provides a normalized measure of the preparatory activity. One-way repeated-measures ANOVAs showed an increased BSI from lower to higher-level areas (Fig. 2C; F(9, 99) = 18.59, *p* < 0.001). This shift was significantly larger than zero in all tested brain areas (*ps* < 0.006; one-sample *t*-test, *FDR-corrected*), except for V1 during motion-based preparatory attention (*p* = 0.081, *FDR-corrected*). These results suggested that higher-level areas actively generate top-down preparatory signals for feature-based selection.

### Preparatory activity patterns contain information about the attended feature but does not rely on sensory template

**Figure 3:**
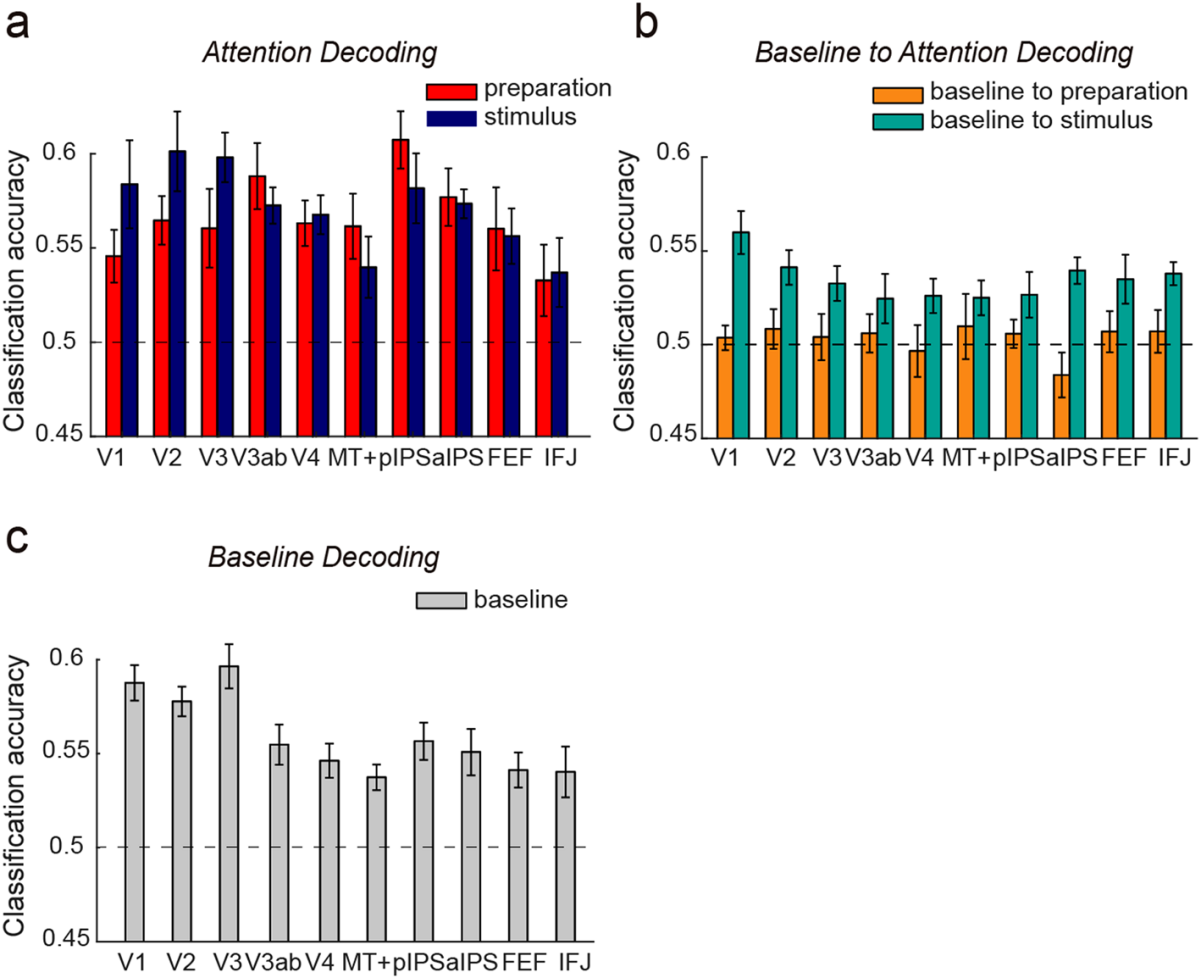
(**a**) Decoding the attended motion direction during preparation (red bars) and stimulus period (blue bars) in individual brain areas. (**b**) Cross-task generalization from baseline task to preparation (yellow bars) and stimulus periods (cyan bars) in the motion-based attention task. (**c**) Decoding the direction of the motion stimulus in the baseline task. The black dashed lines denote theoretical chance level (0.5), which is plotted here for visualization purpose only. Note that the evaluation of the statistical significance of decoding accuracy is based on permutation tests. Error bars denote within-subject errors.

We used MVPA to assess whether the distributed neural pattern for preparatory attention contained feature-specific information. We trained and tested classifiers using data from the preparatory period with a cross-validation approach. Figure 3A showed above-chance classification accuracy for the preparatory period in all tested brain areas (*ps* < 0.02; permutation test, *FDR-corrected*). A similar analysis using data from the stimulus period revealed similar decoding performance (*ps* < 0.005; permutation test, *FDR-corrected*), replicating our prior findings of attentional modulations of stimulus-evoked responses^13,16,42^.

Next, we examined how preparatory activity represents information about the to-be-attended feature. We reasoned that if preparatory attention relies on a sensory template, the activity patterns evoked by preparatory attention should be predictable by the activity patterns from the single feature in the baseline task (“sensory template”). First, we verified that we can use activity patterns in the baseline task to decode a single feature in all brain areas (*ps* < 0.001; permutation test, *FDR-corrected*, Fig. 3C). Next, we conducted a generalization test of the neural patterns from the baseline task to different time periods (preparation and stimulus periods) in the attention task. As shown in Fig. 3B, we did not find reliable generalization from baseline activity to the preparatory activity in any of the tested brain areas (*ps* > 0.458; permutation test, *FDR-corrected*); in contrast to the reliable generalization from baseline activity to stimulus period activity (*ps* < 0.015; permutation test, *FDR-corrected*). These results thus argue against the sensory template hypothesis during preparatory attention and suggest distinct coding formats utilized during preparation and stimulus selection periods, as evidenced by the main effects of time period in cross-task decoding (baseline to preparation vs. baseline to stimulus selection) (F(1, 99) = 19.97, *p* < 0.001; two-way ANOVAs).

As a complementary analysis, we also trained a classifier with neural activity in the stimulus period of the attention task and tested its generalization to the preparatory activity, which again returned chance-level generalization (*ps* > 0.612; permutation test, *FDR-corrected*).

### Spatial attention cannot account for the non-sensory neural signals underlying feature-based preparation

**Figure 4:**
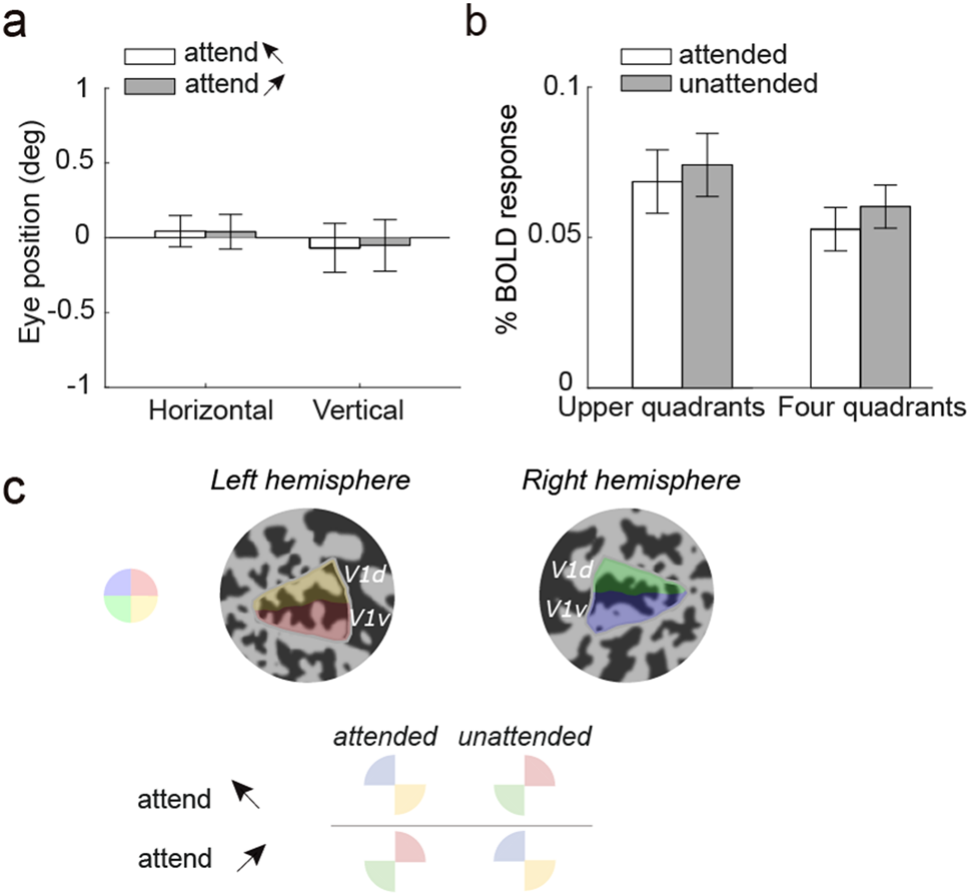
(**a**) Average group-level eye position for the preparation period during behavioral training. (**b**) The averaged BOLD response from the two upper quadrants and all four quadrants for attended vs. unattended locations in V1. Error bars denote within-subject errors. (**c**) A schematic of the quadrant representations in V1. Two flat patches (for the left and right occipital cortex) of a single subject are shown, with quadrants color-coded (upper-left inset shows locations in the visual field). The lower panel depicts the hypothetical scenario in which subjects attended to one of the upper quadrants, or attended to two opposite quadrants when instructed to attend to a specific motion direction.

One possible explanation for the lack of generalization between the baseline task and preparation is that subjects might have used a different attentional strategy during preparatory periods. In particular, instead of using a feature-based strategy, they may have overtly or covertly directed their spatial attention to particular parts of the visual field during preparation (e.g., leftward: upper-left quadrants; Fig. 4C). To assess the potential influence of overt spatial attention in our results, we analyzed the participants’ eye position recorded during the training session and observed no significant difference between attended features during preparation for both features (*p* = 0.501 for horizontal; *p* = 0.604 for vertical; paired *t*-tests; Fig. 4A). Bayes factors indicate moderate evidence for the null hypothesis (BF_01_ = 2.828 for horizontal; BF_01_ = 3.077 for vertical). We note that we only collected eye tracking data during behavioral training and not during scanning due to technical limitations. However, it seems unlikely that the participants would adopt a different eye fixation strategy after training, especially given the centrally presented stimulus configuration that made eye movement an ineffective strategy.

Even though participants likely maintained stable fixation in our experiment, they might have covertly shifted their spatial attention depending on the attended feature. We next assessed whether participants covertly shifted their spatial attention to different quadrants in the visual field during preparation. Such shift should lead to stronger BOLD response when a voxel’s preferred spatial location was attended, especially for early visual cortex such as V1^43,44^. However, BOLD response amplitude (assuming the spatial strategy) during the preparatory period showed no significant difference between the attended and unattended location in V1 (*p* = 0.797 for two upper quadrants and *p* = 0.613 for four quadrants; paired t-tests, Fig. 4B). Bayes factors indicate moderate evidence for the null hypothesis (BF_01_ = 3.376 for upper quadrants and BF_01_ = 3.094 for four quadrants; paired t-tests). Taken together, these control analyses show that neither overt nor covert spatial attention can account for the non-sensory neural representation of preparatory attention.

### Control experiment: verbal coding of attended feature is not utilized during preparatory attention

**Figure 5:**
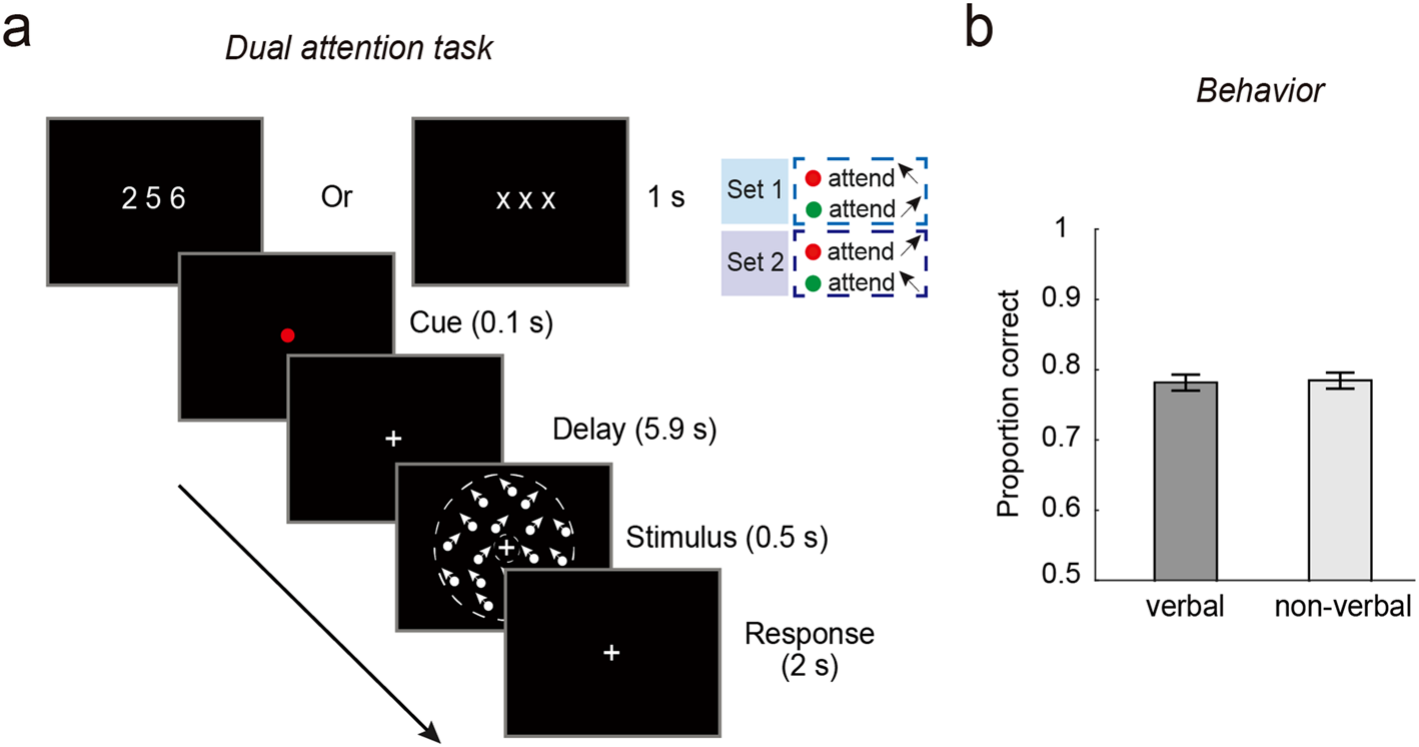
(**a**) Schematic of the articulatory suppression task in the control experiment. The task structure was similar to that in the main experiment (c.f., Fig. 1). At the beginning of each trial, either three digits or “xxx” were shown, indicating whether participants need to read out the digits or not, respectively. (**b**) Task performance, as indexed by the proportion of correct trials. Error bars are within-subject errors.

One possible account for the observed lack of sensory template during preparation is that participants might have used a verbal strategy to maintain information about the cued direction (e.g., repeating the words “leftward” and “rightward” subvocally). To investigate this possibility, we added an articulatory suppression task to disrupt potential verbal rehearsal of the cue-related information throughout the trial. Paired t-test on the discrimination accuracy revealed no significant difference with or without articulatory suppression (*t*(11) = 0.13, *p* = 0.898, Fig. 5). A Bayesian paired t-test returned a Bayes factor of 3.45, providing moderate evidence for the null hypothesis. Thus, the lack of generalization between feature preparation and stimulus selection is unlikely due to different task strategies in the two periods (e.g., verbal vs. visual).

## Discussion

In the present study, we examined how preparatory neural signals convey information about the attended feature in the human brain. Using fMRI and a feature cuing task, we found an elevated pre-stimulus preparatory activity that was stronger in prefrontal and parietal areas than in the early visual cortex, consistent with top-down modulations from these frontoparietal areas in controlling feature-based attention^40,45–48^. Importantly, while decoding of preparatory activity patterns revealed feature-specific information in absence of sensory input, generalization from the baseline task to preparatory attention did not reveal reliable decoding in either visual areas or higher-level frontoparietal areas, in contrast to robust cross-decoding between the baseline task and the stimulus-based attention selection. Our findings thus suggest that preparatory attention is likely dominated by a non-sensory template. In addition, we ruled out the possibility that such a non-sensory template during preparatory periods was due to space-based attentional shifts or verbal re-coding of sensory information.

Because the lack of generalization from the baseline task to preparation is largely a null result, one should be careful in its interpretation. To that end, we have carefully examined multiple alternative possibilities that might explain this result.

First, it might be possible that participants were not actively engaged during the preparatory periods, which would lead to the absence of a sensory template. However, we did observe a significant baseline shift during preparation that was stronger in frontoparietal areas than visual areas (Fig. 2), characteristics of an endogenous, top-down signal. Furthermore, we also observed reliable decoding of to-be-attended feature during preparation (Fig. 3A). Both of these findings speak against the possibility that participants were disengaged from the task during preparation. Second, if participants adopted alternative task strategies, such as shifting their spatial attention or using a verbal strategy during preparation, we may not observe sensory template effects. However, our control analyses and a control experiment speak against this possibility. Third, one may argue that the lack of generalization was due to a low statistical power. We note that the actual generalization accuracy in many brain areas were very close to, or at, the theoretical chance level (0.5, see Fig. 3B). Thus, a higher statistical power is unlikely to help. Furthermore, although our sample size is relatively moderate, it is based on and comparable to many studies using a psychophysical approach to study attention^11,17–19^, including previous studies demonstrating sensory template effects^8,9^. Perhaps most importantly, we were able to decode the attended feature using data from the preparatory period (Fig. 3A), suggesting that there was indeed reliable pattern information during preparatory attention. We also found reliable generalization from baseline to the stimulus period, suggesting that this sample size was sufficient to detect a generalization effect. Overall, we do not believe that any of these alternative possibilities constitute viable explanations of our results. However, to be extremely cautious, we acknowledge that it is still possible that there might be a weak sensory template effect that is beyond the sensitivity of our methods. Yet in the context of other reliable decoding results, our results at least suggest that preparatory attention relies *primarily* on non-sensory representations.

Three potential and non-mutually exclusive explanations may account for the divergence between our results and previous studies supporting sensory template^8–10^. First, these previous studies used familiar objects (letters and natural objects and scenes) while we used a basic visual feature (moving dots). It is possible that preparatory attention for familiar objects more easily evokes sensory templates, perhaps due to a stronger representation in long-term memory. Second, perhaps more critically, there is a subtle methodological difference regarding the meaning of the advanced information at the beginning of the trial (i.e., cue). In previous studies, the cue indicated the object that participants need to detect among noise and distracters, and preparatory activity was extracted from cue-only trials where the target stimulus was expected, but was not physically presented. The cue in our experiments, however, only indicated the relevant feature which was always presented. This difference parallels the distinction between attention and expectation: while attention is deployed based on the task relevance of sensory information, expectation reflects visual interpretations of stimuli due to sensory uncertainty^49–51^. It is known that the latter tends to evoke a sensory template^10,52^, and that attention and expectation can have distinct effects on neural responses^53,54^. Thus, it is possible that previous work on preparatory attention introduced a component of expectation because participants likely expected to perceive the cued object on target-absent trials. Third, task and stimulus variability may also play a role in modulating attentional representations. A sensory template could be more useful when preparing to select targets in a variable context (e.g., visual search in natural images with targets appearing in different locations), compared to the selection of fixed features with subtle variations (as in the current study). Future work is needed to examine the influence of stimulus and task factors during preparatory attention.

The nature of the non-sensory template during preparatory attention needs further investigation. One possibility is that the to-be-attended feature was encoded in a more abstract form, such as a category, during preparation. Previous behavioral studies have hinted at the possibility that feature preparation could occur at a more categorical level. For example, when searching for a target defined by orientation and color, observers appear to implement a search template incorporating categorical information that is not linked to precise physical properties^55,56^. A related possibility, as suggested by recent neuroimaging studies of working memory, is that visual feature is recoded or transformed into alternative forms that deviate from the original sensory template^14,15^. Future studies will be necessary to address the exact nature of non-sensory neural representation underlying preparatory attention.

Taken together, our results suggest that preparatory attention for visual features predominantly relies on a non-sensory template that may reflect an abstraction of visual information. This coding form was evident at different levels of brain areas, indicating a general-purpose mechanism that operates across sensory and association areas. The contrasting findings of sensory vs. non-sensory template during stimulus versus preparatory periods suggest a transformation between different representational formats for attention; such diverse mechanisms likely endow the brain with flexibility during feature-based selection.

## Supplementary Information

Supplementary Information.

## Data Availability

Data and code for the experiment are publicly available at https://osf.io/3s98a/.
