# Supplementary material for: Preparatory attention to visual features primarily relies on non-sensory representation
Source: Sci Rep. 2022 Dec 16;12:21726. doi: 10.1038/s41598-022-26104-2 (PMC9758135; doi:10.1038/s41598-022-26104-2)
Supplement: Supplementary file 1 — Supplementary Information. [file 41598_2022_26104_MOESM1_ESM.pdf]

Supplementary Information

Preparatory attention to visual features primarily relies on non-sensory representation

Abbreviated title: Abstract representation of preparatory attention

Mengyuan Gong[1], Yilin Chen[1], Taosheng Liu[2]

[1]Department of Psychology and Behavioral Sciences, Zhejiang University, Hangzhou 310028, China.

[2]Department of Psychology, Michigan State University, East Lansing, Michigan, 48824.

Corresponding authors email addresses: gongmy426@zju.edu.cn (Mengyuan Gong), tsliu@msu.edu (Taosheng Liu)

Influence of voxel selection on the decoding of attentional signals and cross-task generalization

To examine whether our main findings of attentional decoding and cross-task generalization from the baseline task to the attention task (preparation and stimulus period) were robust to different voxel selection criteria, here, we reported these main results using two additional voxel selection criteria. In the first analysis, we selected the top 60 voxels in each area, ranked by their univariate r2 value (Fig. S1, panels A and B). The decoding of attentional signals was significantly above-chance for across preparatory and stimulus periods in all tested brain areas (*ps* < 0.031, permutation test, *FDR-corrected*). Cross-task generalization analysis revealed significantly above-chance decoding from the baseline to the stimulus period (*ps* < 0.025, permutation test, *FDR-corrected*), but not to the preparation period (*ps* > 0.47, permutation test, *FDR-corrected*). A two-way ANOVA confirmed these differential patterns of generalization tests between two periods ($F(1,99) = 20.08$, $p<0.001$).

In the second analysis, we selected the minimal number of voxels across regions for individual subjects, including on average 102 voxels (SD = 15) (Fig. S1, panels C and D). The same analyses during attention task revealed significantly above-chance decoding performance for both the preparation and stimulus periods in all tested brain areas (*ps*<0.045, permutation test, FDR-corrected), except for a marginal trend in IFJ during the stimulus period (ps = 0.067, permutation test, FDR-corrected). The cross-task generalization was above-chance from the baseline task to the stimulus periods in the attention task (*ps*<0.015, permutation test, FDR-corrected) except for marginal trends in MT+ and IFJ (ps = 0.063, permutation test, FDR-corrected). No significant generalization was found from the baseline to the preparatory period (*ps*>0.36, permutation test, FDR-corrected). Again, a two-way ANOVA confirmed these differential patterns of generalization tests between two periods ($F(1,99)$ =

16.04, p = 0.002). These analyses returned essentially the same results as the main analysis in the paper.

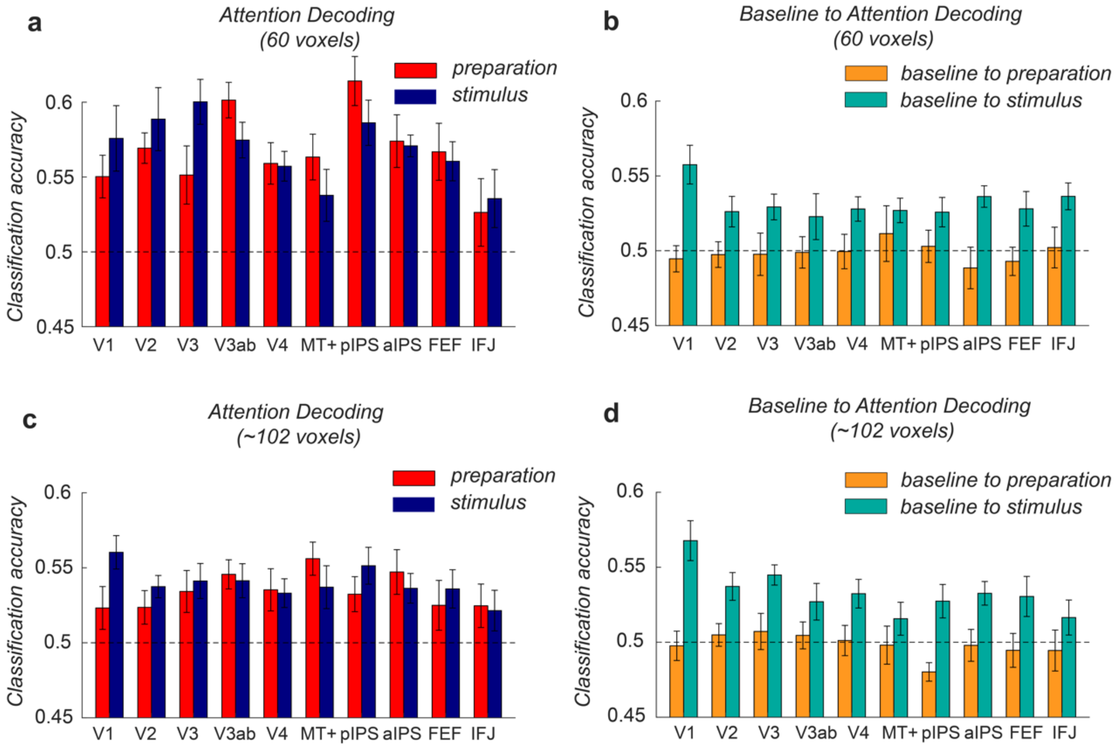

Figure S1. (a) Decoding the attended motion direction during preparation (pink bars) and stimulus period (blue bars) in individual brain areas using 60 voxels. (**b**) Cross-task generalization from baseline task to preparation (pink) and stimulus periods (light blue bars) using 60 voxels. **(c)** The same decoding analysis as that in panel A using individually-defined voxels (on average 102 voxels). **(d)** The same cross-task generalization analysis as that in panel B using individually-defined voxels (on average = 102).
